# A Statistical Physics Approach to Understanding the Adsorption of Methylene Blue onto Cobalt Oxide Nanoparticles

**DOI:** 10.3390/molecules29020412

**Published:** 2024-01-15

**Authors:** Ali Dehbi, Younes Dehmani, Dison S. P. Franco, Hind Omari, Jordana Georgin, Younes Brahmi, Kaoutar Elazhari, Mohammed Messaoudi, Imane Aadnan, Taibi Lamhasni, Awad A. Alrashdi, Abdelaziz Abdallaoui, Sadik Abouarnadasse, Adil Lamini

**Affiliations:** 1 Laboratory of Chemistry/Biology Applied to the Environment, Faculty of Sciences, Moulay Ismail University, Meknes 50070, Morocco; dehbialif@gmail.com (A.D.); omari.hind@gmail.com (H.O.); k.elazhari@gmail.com (K.E.); a.abdallaoui@gmail.com (A.A.); abouarnadasse@yahoo.fr (S.A.);; 2 Department of Civil and Environmental, Universidad de la Costa, CUC, Calle 58 # 55–66, Barranquilla 50366, Colombia; jordanageorgin89@gmail.com; 3 HTMR-Lab, Mohammed VI Polytechnic University (UM6P), Ben Guerir 43150, Morocco; 4 Laboratory of Materials, Membranes and Nanotechnology, Department of Chemistry, Faculty of Sciences, Moulay Ismail University, Meknes 50070, Morocco; mohammed.messeoudi20@gmail.com; 5 Institut National des Sciences de l’Archéologie et du Patrimoine (INSAP), BP 6828, Madinat al Irfane, Avenue Allal El-Fassi, Angle rues 5 et 7, Rabat 10000, Morocco; t.lamhasni@gmail.com; 6 Chemistry Department, Al-Qunfudah University College, Umm Al-Qura University, Mecca 24382, Saudi Arabia

**Keywords:** dyes, methylene blue, cobalt oxide, advanced statistical physics models, logistic models

## Abstract

The production of cobalt oxide nanoparticles and their use in the adsorption of methylene blue (MB) from solution is described in the paper. The X-ray diffraction patterns show that the synthesized cobalt oxide nanoparticles have a crystalline cubic structure. The study of the adsorption of methylene blue onto the cobalt oxide nanoparticles involved determining the contact time and initial concentration of the adsorption of MB on the adsorbent. The kinetics of adsorption were analyzed using two kinetic models (pseudo-first order and pseudo-second order), and the pseudo-second-order model was found to be the most appropriate for describing the behavior of the adsorption. This study indicates that the MLTS (monolayer with the same number of molecules per site) model is the most suitable model for describing methylene blue/cobalt oxide systems, and the parameter values help to further understand the adsorption process with the steric parameters. Indicating that methylene blue is horizontally adsorbed onto the surface of the cobalt oxide, which is bonded to two different receptor sites. Regarding the temperature effect, it was found that the adsorption capacity increased, with the experimental value ranging from 313.7 to 405.3 mg g^−1^, while the MLTS predicted 313.32 and 408.16 mg g^−1^. From the thermodynamic functions, high entropy was found around 280 mg L^−1^ concentration. For all concentrations and temperatures examined, the Gibbs free energy and enthalpy of adsorption were found to be negative and positive, respectively, suggesting that the system is spontaneous and endothermic. According to this study’s findings, methylene blue adsorption onto cobalt oxide nanoparticles happens via the creation of a monolayer, in which the same amount of molecules are adsorbed at two distinct locations. The findings shed light on the methylene blue adsorption process onto cobalt oxide nanoparticles, which have a variety of uses, including the remediation of wastewater.

## 1. Introduction

Industrial activities are a very important source of pollution and contribute in a certain way to the deterioration of the environment. Rejections of the textile industry constitute enormous nuisances for human health, in particular, the various dyes that are used in excess cause wastewater to be highly concentrated with coloring, which has a harmful effect on public health [1,2,3]. Everything in our world is colorful, including food, clothing, cosmetics, medications, and so on. Because of their great range of colors, simplicity of synthesis, and speed of manufacture, these dyes are becoming more and more synthetic in comparison with natural dyes. Today, synthetic dyes are a significant industrial and chemical capital [4,5,6]. The variety of chromophoric groups that make up synthetic dyes, such as azo groups, anthraquinones, triarylmethane, and phthalocyanine, as well as the variety of application technologies (such as reactive, direct, dispersion, and shade reduction) contribute to their structural diversity [7,8,9,10]. The textile strands that make up our clothing are dyed with various colors to achieve their final hue. We cannot even begin to imagine being able to produce similar goods [11]. The textile business mostly uses toxic chemicals, such as some azo dyes that are carcinogenic, during the dyeing and finishing processes, which contaminates surface water and groundwater. It is challenging to reach contamination levels that satisfy environmental standard thresholds following treatment with the used approaches due to the variability in the products used in dyeing [11,12]. Dyes are of great importance in many industrial sectors: they are used for dyeing textiles, paper, etc. But they also cause a lot of damage, especially to the environment and human health [11,12,13]. Furthermore, these compounds are dyes that have a synthetic origin and a complex molecular structure. They are soluble in water, difficult to remove, and generally toxic [11,12,13]. It is therefore necessary to reduce their concentrations in industrial waste as much as possible. Methylene blue is the most common dye used to dye cotton, wool, and silk. It can cause eye burns and permanent injury to the eyes of humans and animals [14,15,16,17]. Its inhalation may cause breathing difficulties, and its ingestion through the mouth produces a burning sensation, causing nausea, vomiting, sweating, and profuse cold sweats [18,19,20]. The treatment of industrial wastes containing this type of dye is of great interest. A wide variety of physical, chemical, and biological techniques have been developed and tested in the treatment of effluents loaded with dyes [21,22,23]. These processes include flocculation, precipitation, ion exchange, membrane filtration, irradiation, and ozonation. However, these procedures are expensive and lead to the generation of large quantities of sludge or the formation of drifts [24,25,26].

Adsorption is the most widely used technique in the treatment of industrial wastewater at the tertiary stage due to its easy implementation and relatively low cost compared with other techniques [1,27,28,29,30,31,32,33]. Adsorption is among the most widely used techniques and is easy to implement. The elimination of dyes in aqueous solutions with adsorption onto various solid materials, particularly transition metal oxides, has been the subject of numerous studies [34,35,36,37]. The adsorption of organic molecules such as those of dyes onto cobalt oxide has proven to be a very effective treatment technique; however, only in the case of certain recalcitrant dyes. Moreover, adsorbent regeneration is an easy and less expensive operation.

In this article, we report on the synthesis of cobalt oxide nanoparticles and their application in the removal of methylene blue from aqueous solutions. The synthesized nanoparticles were characterized with X-ray diffraction, and their morphology was analyzed with scanning electron microscopy. The adsorption process was studied by investigating the effect of initial dye concentration, contact time, and temperature. Kinetic and isotherm models were applied to analyze the adsorption data, and thermodynamic parameters were calculated to determine the feasibility and spontaneity of the adsorption process. The aim of this study was to provide a comprehensive understanding of the adsorption mechanism and to optimize the conditions for the removal of methylene blue using cobalt oxide nanoparticles. The findings of this study have significant implications for the development of effective and sustainable wastewater treatment technologies.

## 2. Results

### 2.1. X-ray Diffraction Pattern Results

**Figure 1:**
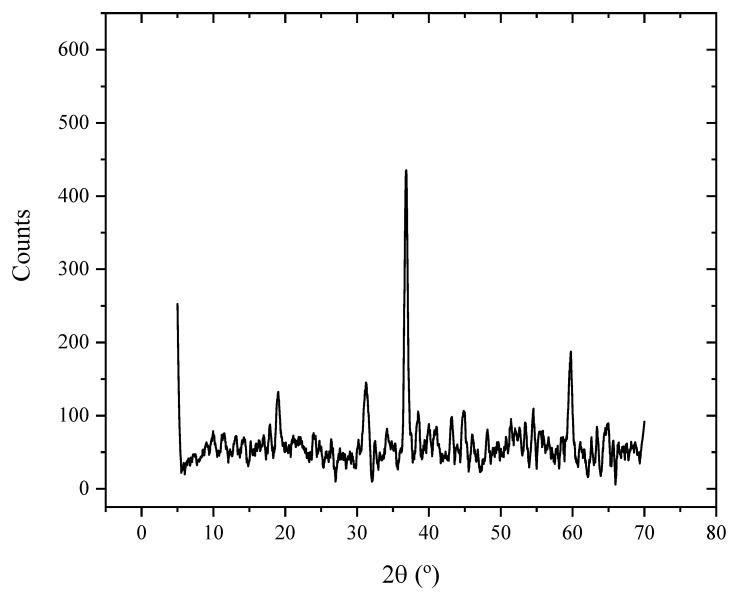
XRD pattern obtained for the cobalt oxide.

Figure 1 displays the cobalt oxide nanoparticles’ X-ray diffraction (XRD). The crystalline nature of the produced Co_3_O_4_ nanoparticles is demonstrated by the extremely strong, conspicuous, and sharp diffraction peaks seen in the XRD pattern lines. The net diffraction peaks of pure Co_3_O_4_ nanoparticles are observed at 2θ values of 31.6°, 37.3°, 44.7°, 59.4°, and 65.2° corresponding to (2 2 0), (3 1 1), (4 0 0), (5 1 1), and (4 4 0), respectively [38,39,40]. All these peaks correspond to cubic Co_3_O_4_ structured according to the JCPDS card number (14-0673) [38,39,40].

### 2.2. FT-IR Results for the Materials

**Figure 2:**
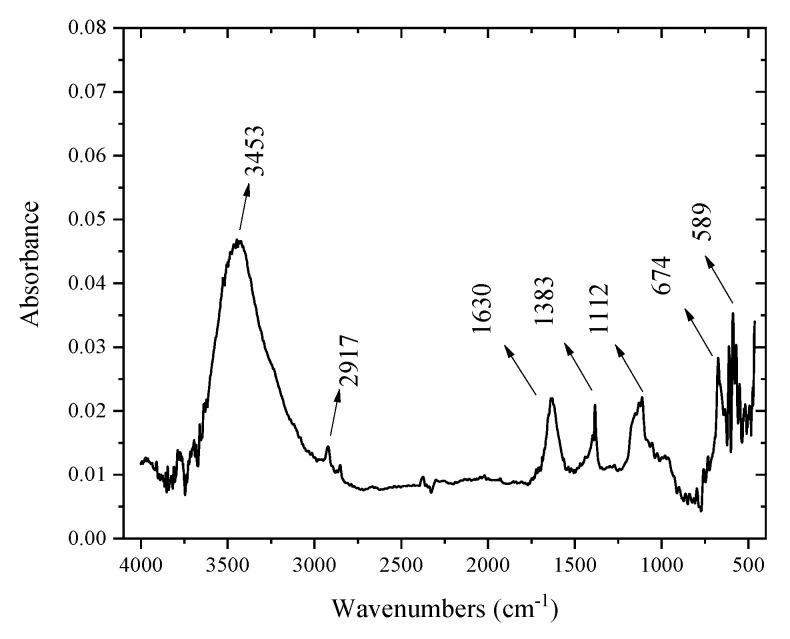
Absorbance according to wavenumber for the cobalt oxide.

Figure 2 displays the prepared solid’s infrared Fourier transform spectrum. All the bands and their attributions are grouped together in Table 1. It can be noted that the bands of the infrared spectra relating to the solids prepared in the presence of ammonia are more intense than those of the solids prepared in the presence of sodium hydroxide. It should also be noted that the bands at 2917, 2851, and 1573 cm^−1^ can be attributed to impurities that come from the air or to the humidity of KBr [38,39,40,41].

### 2.3. SEM Images

**Figure 3:**
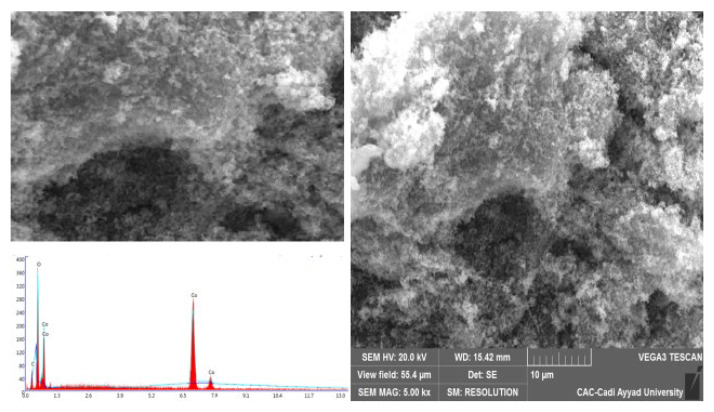
An energy dispersive X-ray (EDX) spectra and a scanning electron microscopy (SEM) picture of the produced cobalt oxide.

Figure 3 shows the scanning electron microscopy image of cobalt oxide prepared using the precipitation method with the precipitating agent ammonia. The image shows a uniform agglomerated morphology. The diffractogram shows the existence of the essential elements of our oxide with well-defined percentages Co (51.97%) and O (48.03%) [38,39,40,41].

### 2.4. Textural Characterization Using the N_2_ Adsorption/Desorption Isotherm

**Figure 4:**
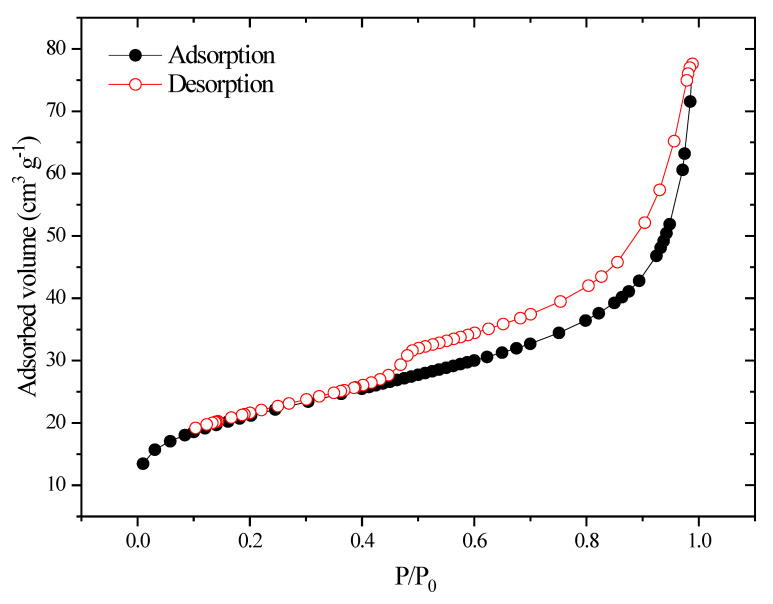
Nitrogen adsorption/desorption isotherms for the cobalt oxide.

Figure 4 illustrates how the IUPAC classification of the nitrogen adsorption/desorption isotherm on cobalt oxide indicates an IV isotherm with an H3 type hysteresis loop, which characterizes meso-porous materials. This solid has a specific surface area on the order of 12 m^2^ g^−1^ with a pore diameter of 167.5270 A and a pore volume on the order of 0.054396 cm^3^/g [38,39,40,41].

### 2.5. Point of Zero Charge

**Figure 5:**
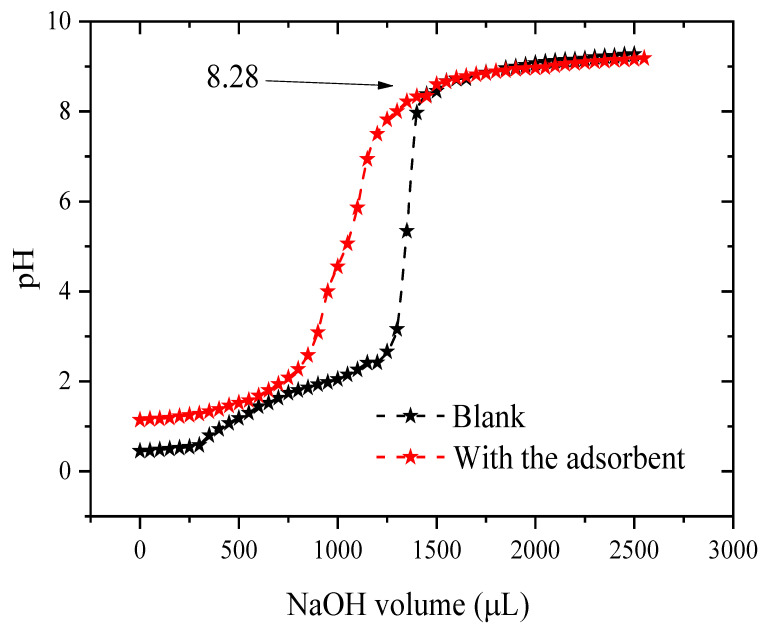
Volumetric titration results both with and without cobalt oxide.

The pH of the zero-charge point corresponds to the pH value for which the net charge on the surface of the adsorbents is zero. This parameter is very important in adsorption phenomena. To determine the zero-charge point, 1 g of cobalt oxide and 100 mL of NaCl (0.001 M) were placed in a beaker and stirred for 24 h to reach the aging time. It was then titrated as follows: 0.5 mL HCl (0.5 M) was added to the NaCl solution, then 50 µL NaOH titration solution (0.2 M) was gradually introduced into the cell, and the adjusted pH values were given for each added volume. The method of blank titration is the same as in the presence of a solid with the same center of gravity as the electrolyte. On a graph of pH = f (Volume of NaOH), the intersection of the curve and the white curve gives the isoelectric point. From Figure 5, it was found that the pHpzc = 8.28. This means that when the pH of the solution is higher than the pHpzc, the dominant charge of the surface will be negative. On the other hand, when the value of the pH solution is lower than pHpzc, the dominant charge of the surface will be positive [42,43,44].

The produced cobalt oxide was characterized using X-ray diffraction (XRD), the Brunauer Emmett Teller (BET) method, and Fourier transform infrared spectroscopy (FT-IR). In summary, the results of these analyses demonstrate that the precipitation method—which uses ammonia hydroxide as a precipitation agent—is a suitable and efficient way to prepare cobalt nanoparticles.

### 2.6. Adsorbent Mass and the Initial pH Effect

**Figure 6:**
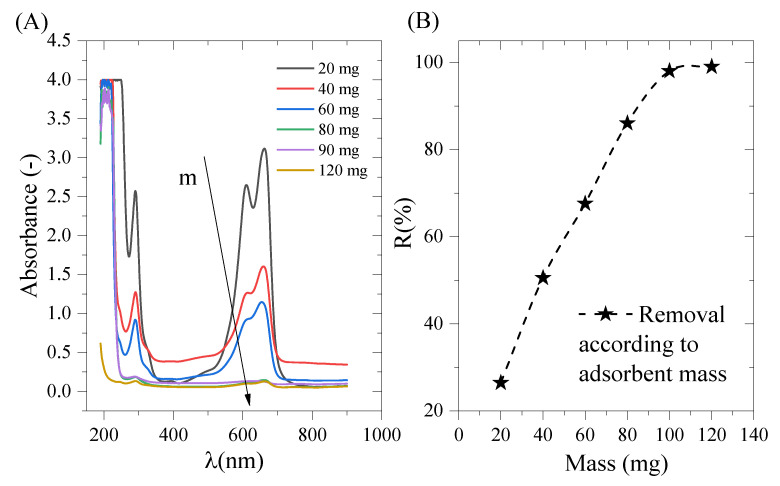
UV-Vis spectrum for methylene blue with different adsorbent masses (**A**) and the percentage of methylene blue removal according to the adsorbent mass (**B**).

The effect of the mass of cobalt oxide on MB adsorption is shown in Figure 6 as an increase in elimination rate as a function of mass. The greater the mass of cobalt oxide, the greater the discoloration of the methylene blue solution [45,46,47,48,49,50]. This behavior is due to the adsorption sites, which increase with the mass of the adsorbent and, therefore, further deplete the methylene blue solution, whose initial concentration is fixed [45,46].

A solution’s initial pH is an important factor in any adsorption study, as it can influence both the adsorbent and adsorbate structure as well as the adsorption mechanism. In the electrostatic interaction between a solid and liquid, a change in pH influences the dominant charges on the surface. In this work, the adsorption efficiency was evaluated by varying the pH from 2 to 12 using a solution of hydrochloric acid HCl (0.1 N) or sodium hydroxide NaOH (0.1 N) according to the desired pH.

**Figure 7:**
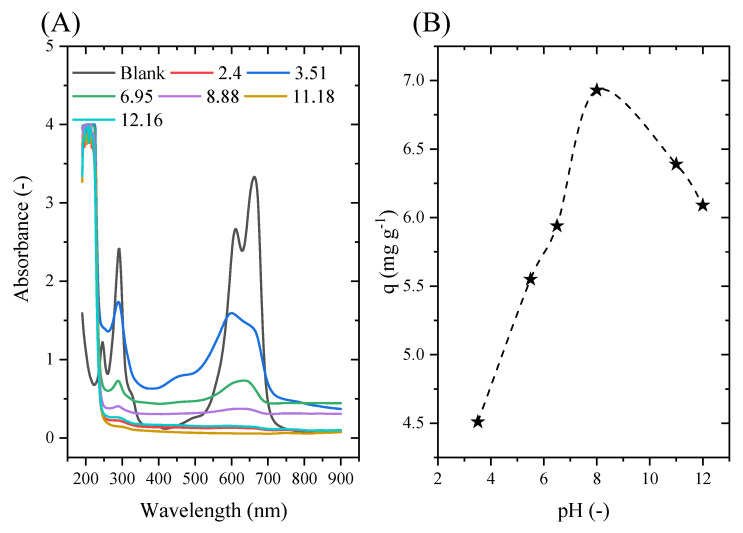
UV-Vis spectrum for methylene blue according to the initial solution pH (**A**) and the adsorption capacity according to the initial solution pH (**B**).

As the pH of the solution rises, Figure 7 demonstrates that more MB is adsorbed. The nature of the dominating charge on the solid surface provides justification for this outcome. The cationic dye molecules in solution have a positive charge, while the surface of the cobalt oxide has a negative charge. Electrostatic interactions between the various charges of the dye and cobalt oxide can be used to conceptualize adsorption [51]. The following explanation explains why the amount of methylene blue adsorbed increases as the pH rises: In an extremely acidic medium, the adsorption of cationic methylene blue is hindered by the neutralization of the cobalt oxide’s negative charge caused by the addition of H+ cations to lower the pH. Additionally, as the pH rises, fewer H+ cations are present, resulting in a negative cobalt oxide charge that facilitates methylene blue adsorption [52]. This can be summed up by saying that at a low pH, H+ ions surround the adsorbent’s surface, which lessens the interaction between the cationic pollutant methylene blue ions and the adsorbent’s sites. However, a high pH causes a drop in H+ concentration, which improves the interaction between the dye’s ions and the surface sites. These findings are consistent with those published in the literature, as previously noted by L.M. Ndjientcheu Yossa [18], who suggested that the optimal pH for methylene blue adsorption is 8.5. Additionally, a pH of 12 was shown to be the ideal pH setting in the study by Kazem Karami [53] and colleagues, with an MB adsorption rate of 97%. Similar to this, Jacob J. Salazar-Rabago’s work [54] revealed the existence of two different forms of methylene blue and showed that electrostatic attraction, particularly at a basic pH, is the main mechanism for MB adsorption with natural white pine sawdust (NS) in aqueous solutions. The discovery that the adsorption of adsorbed MB rises with rising solution pH and falling solution ionic strength supported this conclusion. The electrostatic attraction between the negatively charged surfaces of the cations MB + and NS is responsible for both effects.

### 2.7. Initial Concentration and Adsorption Kinetics

The study of the adsorption of methylene blue on cobalt oxide in solution involves determining the contact time that corresponds to the adsorption/desorption equilibrium or an equilibrium state of saturation of the support by the substrate [46]. The adsorption experiments used to assess the effect of contact time and initial concentration on the adsorption of methylene blue on the adsorbent were performed on solutions of MB in the initial concentration range varying from 0.0001 at 0.001 M and at a temperature of 30 °C for a period of 180 min.

The results obtained show that the adsorbed amount of methylene blue increases rapidly with time in the first 20 min and remains constant after 30 min, indicating a state of equilibrium [47]. This shows that the absorption equilibrium of the dye by the adsorbents used is very fast and can be reached in less than 20 min. Indeed, the remaining unoccupied exterior sites are difficult to occupy, which is due to the formation of repulsive forces between the molecules of methylene blue MB at the surface of the solid and those of the aqueous phase [48]. Note that the initial concentration of MB does not have a significant effect on the equilibrium time, but it does have a significant effect on the adsorption capacity of cobalt oxide. The amount adsorbed increases with an increasing initial concentration of methylene blue [48]. This can be explained by the presence of a large number of molecules that diffuse toward the sites on the surface of the adsorbent; therefore, partial adsorption depends on the initial concentration [49,50].

**Figure 8:**
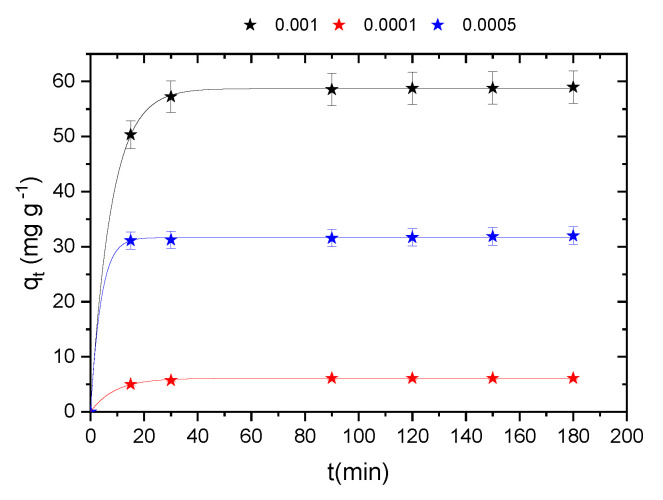
Effect of the initial methylene concentration on the adsorption kinetics and best-fitted model for the adsorption of methylene blue onto cobalt oxide.

The kinetic study of MB adsorption was based on the analysis of kinetic curves (Figure 8) according to two kinetic models (pseudo-first order and pseudo-second order). The comparison between the values q_e_ and R^2^ for the models can help in determining the best-suited model. The R^2^ values of the pseudo-second-order kinetic model are higher than those of the pseudo-first-order kinetic model, and the values of q_e_ calculated using the pseudo-second-order kinetic model are also much closer to q_e,exp_ than those of the model pseudo-first-order kinetics (Table 2). Therefore, the pseudo-second-order kinetics model is more appropriate for describing the behavior of the adsorption of methylene blue onto cobalt oxide.

### 2.8. Isotherm Results and Physical–Statistical Modeling

**Figure 9:**
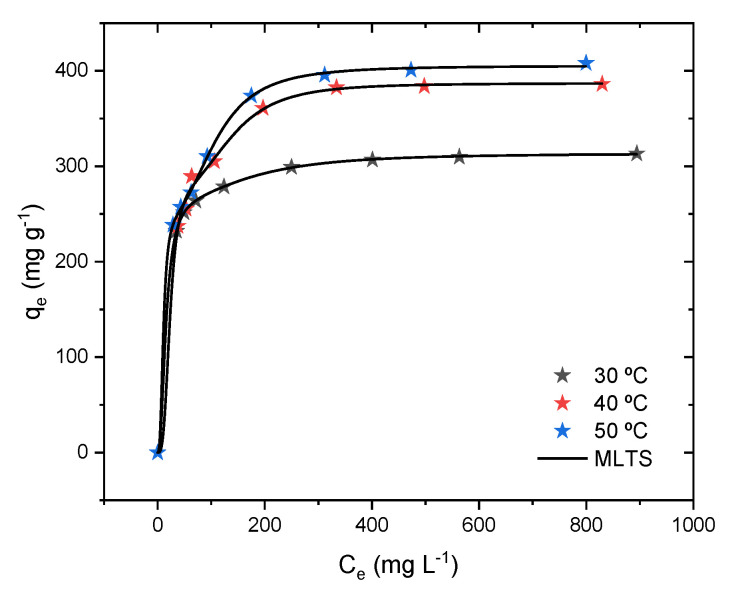
Adsorption isotherms for methylene blue onto the cobalt oxide for different temperatures showing the best-fitted physical–statistical model.

The adsorption isotherms were conducted to understand the behavior of methylene blue at the equilibrium of the solid and liquid phases at different temperatures, here shown in Figure 9. The first aspect is that the adsorption capacity trends to increase according to the concentration at equilibrium, with the formation of a plateau after 200 mg L^−1^, despite the system temperature. The second effect to take into consideration is the temperature of the system, which indicates that the adsorption capacity increases with the variation in the temperature. In this case, the cobalt oxide was able to be adsorbed, in terms of the maximum experimental adsorption capacity, at 313.3, 386.1, and 408.1 mg g^−1^ according to the system temperatures of 30, 40, and 50 °C. This means that increasing the temperature of the system gives more energy, which translates into an increase in the adsorption capacity. This can be related to an increase in the movement of the molecules, which enables methylene blue to reach adsorption sites that were previously unattainable.

Aiming to elucidate the adsorption of methylene blue onto the cobalt oxide, the physical–statistical models were fitted to the experimental data. The best model is shown in Figure 9. In this case, it was found that the MLTO is the most suitable model for describing the methylene blue/cobalt oxide systems. Although the other fitted models present good statistical indicators, the parameter tendency does not reflect the tendency of the experimental data, meaning that the concentration at half-saturation increased when it should have decreased (see Tables S1 and S2 in the Supplementary Materials) [55]. The MLTO indicates that adsorption occurs with the formation of the monolayer, where the same number of molecules are adsorbed onto two different sites. This result is in agreement with the kinetical results, where the PSO model was the most suitable, meaning that the methylene blue molecules need activated sites to be adsorbed. As mentioned in the modeling section, the PSMs are important models, as the parameter values help to further understand the adsorption process based on the steric parameters, which are further discussed.

**Figure 10:**
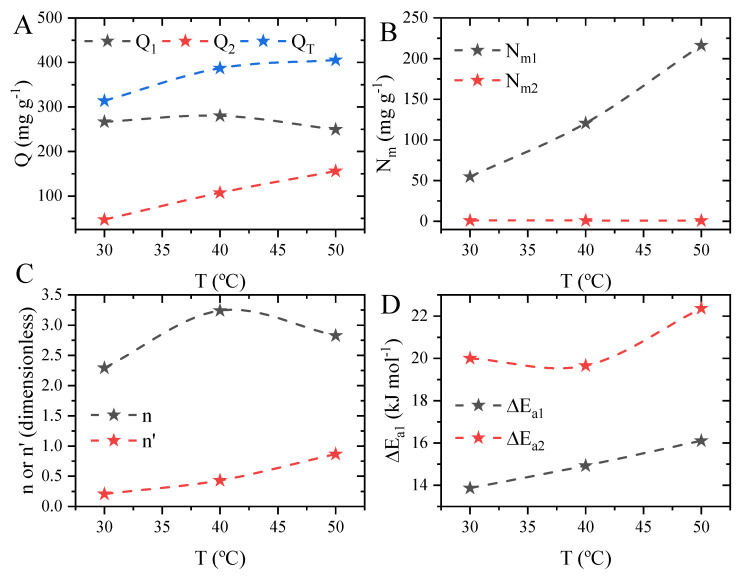
PSM steric parameters obtained for the adsorption of methylene blue onto cobalt oxide according to the system temperature: adsorption capacity (**A**), receptor density (**B**), number of molecules per site, anchorage number (**C**), and adsorption energy (**D**).

The steric parameters are the number of molecules adsorbed per site (n, dimensionless), in this case, par of sites, the density of the adsorption site (N_m_, mg g^−1^), and the concentration at half-saturation (C_1_ and C_2_, mg L^−1^), which are used for the determination of the adsorption energy (∆E_a_, kJ mol^−1^) [22]. The estimated parameters and the maximum adsorption capacity of the model are shown in Figure 10. The first aspect is the total adsorption capacity predicted with the MLTO (Figure 10A) to be 313.70, 387.12, and 405.31 mg g^−1^, which is close to the experimental value of 313.32, 386.17, and 408.16 mg g^−1^. However, it should be taken into consideration that the total sum of the adsorption capacity is obtained from the adsorption capacity of each site, and it was found that the adsorption capacity of the first site (Q_m1_) tends to oscillate according to the temperature system, going from 266.8 to 280.0 and then to 249.4 mg. As for the second site, it was found that the adsorption capacity tends to increase linearly with the system temperature, going from 46.95 to 155.9 mg g^−1^. This indicates that the first type of site may suffer some change at the highest temperature (50 °C), which diminishes the adsorption.

It is also possible to obtain the density of the adsorption sites, as shown in Figure 10B. The first aspect to note is that the density of the first receptor site increases linearly with the temperature of the system, while the density of the second site tends to remain constant over the temperature range. The main explanation for this is the oscillation in the number of methylene blue molecules captured by the adsorbent [56]. Furthermore, the behavior of the receptor density may be related to the composition of the cobalt oxide or another possible mechanism. This means that the presence of the NaOH (used for the pH change) can cause the substitution of the hydroxyl groups onto oxygen groups present in the material forming CoOH, which can lead to a change in the material and the surface charge [57]. Considering that receptors 1 and 2 can be associated with the quantity of each major group, in this case, the CoO and CoOH should be receptor sites 1 and 2, respectively.

The number of molecules per site is an important parameter that indicates how the molecules are adsorbed at the surface of the adsorbent, meaning that when the n values are above the unit (1), the molecules will tend to be adsorbed in a “horizontally” manner; on the other hand, when the values are under the unit, the molecules are adsorbed in a “parallel” way. In this case, the MLTO considers that the same number of molecules are adsorbed in both sites with different energy, as shown in Figure 10C [58]. The first aspect to note is that the number of molecules per site tends to oscillate according to the system temperature: 2.29, 3.24, and 2.825. However, it should also be noted that the number of molecules is above the unit for all cases, which means that all the methylene blue molecules are adsorbed in a horizontal manner in relation to the surface of the material. Furthermore, the number of molecules per site can be used to determine the anchorage number (n′ = 1/n), which corresponds to the occupation of the receptor sites. In this case, it was found that despite the temperature of the system, the methylene blue will not occupy a complete site of receptors 1 or 2 [59].

Lastly, the adsorption energy was obtained for both receptors according to the system temperature, as shown in Figure 10D. The first aspect to note is that the energy adsorption values were below 40 kJ mol^−1^, which directly indicates that all the interactions that occur between the methylene blue and the cobalt oxide are due to physics interactions [60], which are further described in the proposal of the adsorption mechanism. In the second aspect, it was found that the adsorption energy of the first receptor is lower than that of the second, meaning that methylene blue is more easily adsorbed onto the first receptor site in comparison with the second one. Regarding the temperature of the effect, it was found that the adsorption energy tends to increase for both receptors: for the first one, it goes from 13.87 to 16.20 kJ mol^−1^, while for the second one, it goes from 20.00 to 22.36 kJ mol^−1^ according to the system temperature.

### 2.9. Potential Thermodynamic Functions and Thermodynamic Simulations

Using the best-fitted physical–statistical model, it is possible to obtain thermodynamic results with the application of potential functions. The potential functions are related to the configuration entropy (S_a_, kJ mol^−1^ K^−1^), Gibbs free energy of adsorption (G_a_, kJ mol^−1^), internal energy of adsorption (E_int_, kJ mol^−1^), and enthalpy of adsorption (H_a_, kJ mol^−1^) [60].

(1) $$\frac{S_{a}}{k_{B}}=\ln\left(Z_{\mathrm{gc}}\right)-\beta \frac{\partial }{\partial \beta }\ln\left(Z_{\mathrm{gc}}\right)$$

(2) $$G_{a}=\mu Q_{0}$$

(3) $$\mu =\frac{1}{\beta }\ln\left(\frac{C_{e}}{{\left(\frac{2\pi m}{h^{2}\beta }\right)}^{3/2}}\right)$$

(4) $$E_{\mathrm{int}}=\frac{\mu }{\beta }\left(\frac{\partial }{\partial \mu }\ln\left(Z_{\mathrm{gc}}\right)\right)-\frac{\partial }{\partial \beta }\ln\left(Z_{\mathrm{gc}}\right)$$

(5) $$H_{a}=G_{a}-TS_{a}$$

where Z_gc_ represents the total grand canonical partition function, μ denotes the chemical potential of the adsorbed molecule (in units of J mol^−1^), β is expressed as the constant equal to 1/k_B_T, where k_B_ is the Boltzmann constant (1.380649 × 10^−23^ J K^−1^), T is the temperature (in K), h represents the Planck constant (6.62607004 × 10^−34^ m^2^ kg s^−1^), and m stands for the mass of the adsorbate (112,411 mg mol^−1^ for methylene blue). From the MLTS derivation step, it is possible to obtain the partial derivative of the grand canonical ensemble in relation to the constant β and chemical potential µ, as presented by the following equations:

(6) $$\frac{S_{a}}{k_{B}}=\left\{\begin{array}{c} N_{m1}\left[\;\ln\left(1+{\left(\frac{C_{e}}{C_{1}}\right)}^{n}\right)-\ln\left(1+{\left(\frac{C_{e}}{C_{1}}\right)}^{n}\right)\frac{{\left(\frac{C_{e}}{C_{1}}\right)}^{n}}{1+{\left(\frac{C_{e}}{C_{1}}\right)}^{n}}\right]\\ +N_{m2}\left[\ln\left(1+{\left(\frac{C_{e}}{C_{2}}\right)}^{n}\right)-\;\ln\left(1+{\left(\frac{C_{e}}{C_{2}}\right)}^{n}\right)\frac{{\left(\frac{C_{e}}{C_{2}}\right)}^{n}}{1+{\left(\frac{C_{e}}{C_{2}}\right)}^{n}}\right] \end{array}\right.$$

(7) $$G_{a}\beta =\ln\left(\frac{C_{e}}{{\left(\frac{2\pi m}{h^{2}\beta }\right)}^{3/2}}\right)\left(\frac{Q_{m1}}{1+{\left(\frac{C_{1}}{C_{e}}\right)}^{n}}+\frac{Q_{m2}}{1+{\left(\frac{C_{2}}{C_{e}}\right)}^{n}}\right)$$

(8) $$E_{\mathrm{int}}=\left\{\begin{array}{c} {\;N}_{m1}\left[\mu \frac{{\left(\frac{C_{e}}{C_{1}}\right)}^{n}}{1+{\left(\frac{C_{e}}{C_{1}}\right)}^{n}}\frac{1}{\beta }\ln\left(1+{\left(\frac{C_{e}}{C_{1}}\right)}^{n}\right)\frac{{\left(\frac{C_{e}}{C_{1}}\right)}^{n}}{1+{\left(\frac{C_{e}}{C_{1}}\right)}^{n}}\right]\\ +N_{m2}\left[\mu \frac{{\left(\frac{C_{e}}{C_{2}}\right)}^{n}}{1+{\left(\frac{C_{e}}{C_{2}}\right)}^{n}}-\frac{1}{\beta }\ln\left(1+{\left(\frac{C_{e}}{C_{2}}\right)}^{n}\right)\frac{{\left(\frac{C_{e}}{C_{2}}\right)}^{n}}{1+{\left(\frac{C_{e}}{C_{2}}\right)}^{n}}\right] \end{array}\right.$$

(9) $$H_{a}=\left\{\begin{array}{c} ln\left(\frac{C_{e}}{{\left(\frac{2\pi m}{h^{2}\beta }\right)}^{3/2}}\right)\left(\frac{Q_{m1}}{1+{\left(\frac{C_{1}}{C_{e}}\right)}^{n}}+\frac{Q_{m2}}{1+{\left(\frac{C_{2}}{C_{e}}\right)}^{n}}\right)\\ -Tk_{B}\left\{\begin{array}{c} N_{m1}\left[\;\ln\left(1+{\left(\frac{C_{e}}{C_{1}}\right)}^{n}\right)-\ln\left(1+{\left(\frac{C_{e}}{C_{1}}\right)}^{n}\right)\frac{{\left(\frac{C_{e}}{C_{1}}\right)}^{n}}{1+{\left(\frac{C_{e}}{C_{1}}\right)}^{n}}\right]\\ +N_{m2}\left[\ln\left(1+{\left(\frac{C_{e}}{C_{2}}\right)}^{n}\right)-\;\ln\left(1+{\left(\frac{C_{e}}{C_{2}}\right)}^{n}\right)\frac{{\left(\frac{C_{e}}{C_{2}}\right)}^{n}}{1+{\left(\frac{C_{e}}{C_{2}}\right)}^{n}}\right] \end{array}\right. \end{array}\right.$$

**Figure 11:**
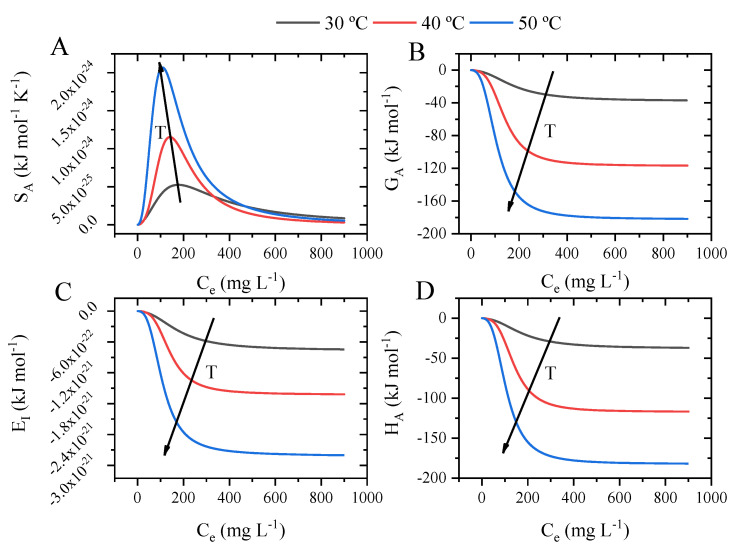
Thermodynamic variable changes for the adsorption of methylene blue onto cobalt oxide: change in configuration entropy (**A**), change in Gibbs free energy (**B**), change internal energy (**C**), and change in adsorption enthalpy (**D**).

The simulation results are given in Figure 11, where each thermodynamic state’s variables are given concerning the equilibrium concentration and temperature of the system. The change in the configuration entropy is shown in Figure 11A, where the entropic curves show similar behavior, with the presence of an entropic peak between 150 and 200 mg L^−1^, which corresponds to the concentration at half-saturation. After this point, the entropy starts to decrease until it reaches equilibrium above 800 mg L^−1^. This means that after the concentration at half-saturation, the methylene blue tends to quickly organize at the surface of the cobalt oxide. The simulation for the configurational Gibbs energy is given in Figure 11B. The first aspect to note is that the Gibbs energy tends to become more negative according to the concentration at equilibrium and temperature, reaching maximum values around the same concentration region as the configuration entropy. This behavior is directly related to an increase in the movement of the methylene blue at the concentration of half-saturation. The evolution of the internal energy according to temperature is given in Figure 11C. It was found that the E_i_ values tend to be more negative according to the system temperature, which indicates that it is more spontaneous. Lastly, the configuration enthalpy is shown in Figure 11D. It was found that the adsorption is endothermic, corroborating the results found for the adsorption energy. In addition, the enthalpy value changes according to the system temperature and concentration, reaching a maximum value of −160 kJ mol^−1^ and indicating that methylene blue is adsorbed due to physical interactions until 200 kJ mol^−1^ [61].

### 2.10. Adsorption Mechanism Proposal

**Figure 12:**
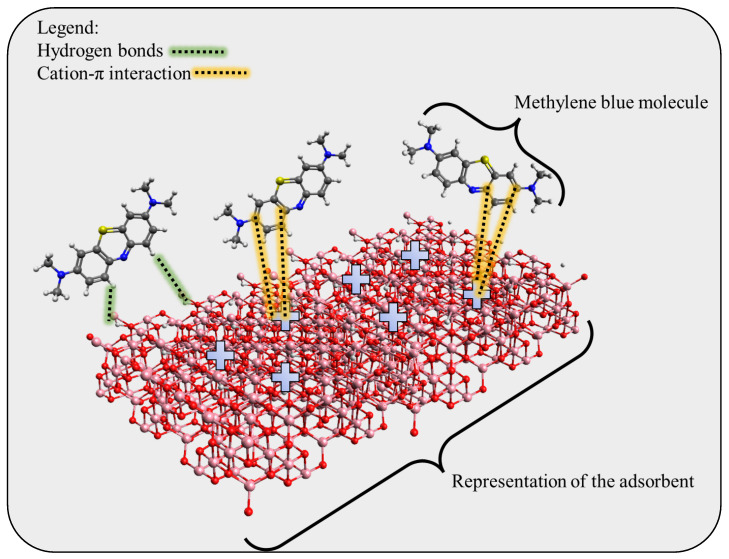
Proposed adsorption mechanism for the methylene blue and cobalt oxide.

An adsorption mechanism can be proposed by considering the characterization stage results, the pH of the solution, the methylene blue speciation, and the physical and statistical data. It is important to keep in mind that cobalt oxide (CoO) makes up the majority of the material when it comes to the adsorption composition. The pH_PZC_ of cobalt oxide is 8.5. This number attests to the cobalt oxide surface’s alkaline character. The pH at which the adsorbent’s surface charge density is neutral is known as the PZC pH. The surface charge is positive when the pH of the solution is lower than the pH_PZC_ and negative when the pH is greater than the pH_PZC_. This demonstrates how closely the surface charge and functional groups of cobalt oxide are related. Because the pH of the solution influences both the shape of MBs in solution and the distribution of surface charges on the biomaterials, the adsorption capacity of cobalt oxide to MBs is primarily dependent on the pH of the solution. Stated differently, pH has the potential to affect the attraction or repulsion of electrostatic interactions between the oxide surface and the MB species found in the solution. This demonstrates how closely the surface charge and functional groups of cobalt oxide are related. Because the pH of the solution influences the shape of MBs in the solution as well as the distribution of surface charges on the biomaterials, the adsorption capacity of cobalt oxide to MBs is primarily dependent on pH. Put differently, pH can affect how repulsive or attractive the electrostatic interactions are between the oxide surface and the MB species that are present in a solution. Regarding methylene blue, we are aware that it exists in two distinct states: at pH 8, all the molecules are in the cationic form (for more on molecular states and speciation, see the Supplementary Materials). Given the results observed and the description given here, it is reasonable to assume that the presence of hydroxyl groups will result in a positive charge on the material’s surface. Therefore, the primary adsorption mechanism is possible hydrogen bonds and the cation-π interaction, as seen in Figure 12.

## 3. Materials and Methods

### 3.1. Chemicals

All chemicals were of analytical grade and used directly without further purification. Cobalt nitrate hexahydrate (Co(NO_3_)_3_, 6H_2_O, 99%), methylene blue (MB, 99%), ammonium hydroxide solution (NH_4_OH, >25% NH_3_), sodium hydroxide (NaOH, 99%), and hydrochloric acid (HCl, 99%) were purchased from Sigma-Aldrich (St. Louis, MO, USA). Distilled water with a specific conductivity of approximately 0.5 μS cm^−1^ was used for all experiments.

### 3.2. Preparation of Co_3_O_4_ and Its Characterization

Cobalt nitrate and ammonium hydroxide were used as precursors or precipitants. First, 1 mass of Co(NO_3_)2.6H_2_O was dissolved in 120 mL of distilled water. Then, 20 mL of a molar NH_4_OH solution was added dropwise (7 mL/min) into the mixture. The final solution was heated to 40 °C and stirred for 1 h. After vacuum filtration, the resulting solid was washed several times with distilled water and dried in an oven at 100 °C overnight. The Co_3_O_4_ powders were heat-treated at 450 °C for 3 h.

The crystal structure of the ZnO particles was analyzed using a Bruker AXS D8 ADVANCE X-ray diffractometer equipped with an X-ray tube (Cu-Kα radiation: λ = 1.541838 Å, 40 kV, 40 mA). Measurements were performed at room temperature over a range of 2–70° (2θ) with a step size of 0.02° and a scan rate of 3 s/step.

FTIR spectra were recorded with a Jasco FTIR 4100 spectrometer (Oklahoma City, OK, USA) using KBr particles with a spectral range of 400–4000 cm^−1^ and a spectral resolution of 4 cm^−1^, performing at least 64 scans per sample.

SEM images were acquired using a Quanta 200 EIF equipped with standard secondary electron (SE) and backscattered electron (BSE) detectors. The electron beam was generated with a conventional tungsten electron source, which can resolve structures down to 3 nm under optimal operating conditions. The microscope was equipped with an Oxford Inca energy dispersive X-ray (EDX) system for elemental chemical analysis.

### 3.3. Adsorption Kinetics and Isotherms for the Methylene Blue

The kinetic study of discontinuous adsorption of methylene blue on a cobalt oxide was carried out by mixing 100 mg of adsorbent with 20 mL of MB solution, with an initial concentration C_0_ varying according to the experiment (Coi 10^−4^; 5 × 10^−4^; 10^−3^ M) in 30 mL Erlenmeyer flasks. The mixture was stirred for a contact time t at a constant pH (pH = 8) and temperature. The MB adsorption isotherm on the adsorbent was carried out under the same experimental conditions by increasing the initial concentrations (C_0_) of MB from 0.0001 to 0.001 M. After each adsorption test, the solid was separated from the liquid phase using a centrifuge at 3000 rpm for 20 min. The residual concentration C_e_ of MNP was determined from the calibration line, fixed at the maximum absorption of MB at λ = 664 nm. The measurements were made with a Shimadzu UV/Visible spectrophotometer (Shimadzu, Kyoto, Japan, UV-1240). MB uptake on each adsorbent was calculated using the following equation [62]:

(10) $$Q_{\mathrm{ads}}=\frac{\left(C_{0}-C_{e}\right)}{m_{\mathrm{ads}}}\times V_{\mathrm{sol}}$$

(11) $$R=\frac{\left(C_{0}-C_{e}\right)}{C_{0}}\times 100\%$$

where Q_ads_ is the adsorption capacity (mg g), C_0_ is the initial concentration of methylene blue (mg L^−1^), C_e_ is the residual concentration of methylene blue (mg L^−1^), mads is the mass of cobalt oxide (g), and V_sol_ is the volume of the methylene blue solution (L).

### 3.4. Kinetics Modeling

The experimental data were evaluated with the application of traditional kinetic models, namely, the pseudo-first- and pseudo-second-order models [63,64]

(12) $$q_{t}=q_{q}(1-e^{-tk_{1}})$$

(13) $$q_{t}=q_{e}^{2}k_{2}\frac{t}{q_{e}k_{2}t+1\;}$$

where q_t_ is the methylene blue adsorption capacity according to time (mg g^−1^), q_e_ is the methylene blue adsorption capacity at equilibrium (mg g^−1^), k_1_ is the pseudo-first-order kinetic constant (min^−1^), k_2_ is the pseudo-second-order kinetic constant (g mg^−1^ min^−1^), and t is time (min).

### 3.5. Isothermal Modeling

Aiming to give another interpretation regarding the adsorption isotherms, we chose to use physics–statics models (PSMs). Each model is based on the grand canonical ensemble and can lead to different information regarding the solid/liquid system. PSMs can be classified according to different proprieties, such as the number of energy sites, the number of molecules adsorbent per site, or the number of layers formed by the adsorbate molecule. In this case, the monolayer model with one energy site (MLO) [65], with two energy sites, and the same number of adsorbed molecules (MLTS) [64,65,66,67], with two energy sites and a different number of adsorbed molecules (MLTD) [21] was chosen, here presented as Equations (5), (6), and (7), respectively. In addition to that, dual-layer models were also used, including the dual-layer model with one energy site (DLO) and with two energy sites (DLT), as depicted in Equations (8) and (9) [66]:

(14) $$q_{e}=\frac{{\mathrm{nN}}_{m}}{1+{\left(\frac{C_{1/2}}{C_{e}}\right)}^{n}}=\frac{q_{m}}{1+{\left(\frac{C_{1/2}}{C_{e}}\right)}^{n}}$$

(15) $$q_{e}=\frac{n_{1}N_{m1}}{1+{\left(\frac{C_{1}}{C_{e}}\right)}^{n}}+\frac{n_{2}N_{m2}}{1+{\left(\frac{C_{2}}{C_{e}}\right)}^{n}}=\frac{q_{m1}}{1+{\left(\frac{C_{1}}{C_{e}}\right)}^{n}}+\frac{q_{m2}}{1+{\left(\frac{C_{2}}{C_{e}}\right)}^{n}}$$

(16) $$q_{e}=\frac{n_{1}N_{m1}}{1+{\left(\frac{C_{1}}{C_{e}}\right)}^{n_{1}}}+\frac{n_{2}N_{m2}}{1+{\left(\frac{C_{2}}{C_{e}}\right)}^{n_{2}}}=\frac{q_{m1}}{1+{\left(\frac{C_{1}}{C_{e}}\right)}^{n_{1}}}+\frac{q_{m2}}{1+{\left(\frac{C_{2}}{C_{e}}\right)}^{n_{2}}}$$

(17) $$q_{e}={\mathrm{nN}}_{m}\frac{{\left(\frac{C_{e}}{C_{1/2}}\right)}^{n}+2{\left(\frac{C_{e}}{C_{1/2}}\right)}^{2n}}{1+{\left(\frac{C_{e}}{C_{1/2}}\right)}^{n}+{\left(\frac{C_{e}}{C_{1/2}}\right)}^{2n}}=q_{m}\frac{{\left(\frac{C_{e}}{C_{1/2}}\right)}^{n}+2{\left(\frac{C_{e}}{C_{1/2}}\right)}^{2n}}{1+{\left(\frac{C_{e}}{C_{1/2}}\right)}^{n}+{\left(\frac{C_{e}}{C_{1/2}}\right)}^{2n}}$$

(18) $$q_{e}={\mathrm{nN}}_{m}\frac{{\left(\frac{C_{e}}{C_{1}}\right)}^{n}+2{\left(\frac{C_{e}}{C_{2}}\right)}^{2n}}{1+{\left(\frac{C_{e}}{C_{1}}\right)}^{n}+{\left(\frac{C_{e}}{C_{2}}\right)}^{2n}}=q_{m}\frac{{\left(\frac{C_{e}}{C_{1}}\right)}^{n}+2{\left(\frac{C_{e}}{C_{2}}\right)}^{2n}}{1+{\left(\frac{C_{e}}{C_{1}}\right)}^{n}+{\left(\frac{C_{e}}{C_{2}}\right)}^{2n}}$$

where n is the number of molecules per site (1 and 2 for the models with two different energy sites), N_m_ is the density of the receptor of the adsorbent (mg g^−1^), q_m1_ is the predicted adsorption capacity according to the model (mg g^−1^), and C_1/2_, C_1_, and C_2_ are the concentrations at the half-saturation (mg L^−1^).

### 3.6. Parameter Estimation and Model Evaluation

Calibration and evaluation of the models are critical steps in adsorption studies. In this case, a Matlab script was used for the calibration and evaluation of the parameters using statistical indicators. An initial guess for each parameter was performed using the particle swarm function (particleswarm). To determine the final parameter value, (lsqnonlin) was used for the models with parameter restriction, and (nonlinfit) was used for the model without parameter restriction. The parameter indicators were obtained after fitting the models, as described in the Supplementary Materials.

## 4. Conclusions

To sum up, the research discussed in this article concentrated on creating cobalt oxide nanoparticles and using them to remove methylene blue (MB) from solutions. The produced cobalt oxide nanoparticles have a crystalline cubic structure, according to the results. Two kinetic models were used to study adsorption kinetics, and it was discovered that the pseudo-second-order model best captured the adsorption behavior. The research results also indicated that the best model to describe the methylene blue/cobalt oxide systems is the MLTS (monolayer with same number of molecules per site) model. It was discovered that the system is spontaneous and endothermic and that the adsorption capacity rises with temperature. According to this study’s findings, methylene blue adsorption onto cobalt oxide nanoparticles happens with the creation of a monolayer, in which molecules are adsorbed onto two distinct sites in equal amounts. These results offer insightful information about the methylene blue adsorption process on cobalt oxide nanoparticles, which has a variety of uses, including the treatment of wastewater.

## Figures and Tables

**Table 1:** The bands and types of vibration for the solid.

Bands (cm^−1^)	Types of Vibration
3453	H–O–H
1630	H–O–H
1383	O=C=O
1112	H–O–H
674	Co–O
609	Co–O
589	Co–O

**Table 2:** Estimated parameters for the kinetical models.

Kinetic Model	Parameters	Concentration (M)		
		10^−3^	5 × 10^−4^	10^−4^
	Q_exp_ (mg g^−1^)	58.99	31.98	6.12
Pseudo-first order	k_1_ (min^−1^)	11.69	6.73	1.69
	q_e_ (mg g^−1^)	57.11	31.57	5.86
	R^2^	0.97	0.99	0.96
Pseudo-second order	k_2_ (g mg^−1^ min^−1^)	0.0064	0.079	0.043
	q_e_ (mg g^−1^)	60	31.84	6.30
	R^2^	0.99	0.99	0.99

## Data Availability

Data are contained within the article and Supplementary Materials.

## Supplementary Materials

The following supporting information can be downloaded at: https://www.mdpi.com/article/10.3390/molecules29020412/s1.
