# Supplementary material for: A Statistical Physics Approach to Understanding the Adsorption of Methylene Blue onto Cobalt Oxide Nanoparticles
Source: Molecules. 2024 Jan 15;29(2):412. doi: 10.3390/molecules29020412 (PMC10818394; doi:10.3390/molecules29020412)

Supplementary Material

S1. Derivation of the physics statistics equations

In this section, a brief derivation of each physical statistical model employed in this work is shown. For the monolayer model with one energy site (M_1), the following formalization is given: i) it is considering that a variable number of molecules N are adsorbed onto N_m receptor sites; ii) the receptor site is supposed to be empty or occupied by one or more molecules, being represented by N_i (0 being empty and 1 being occupied); iii) the grand canonical partition function is used to describe the phenomena for one site:

$$z_{gc} = \sum_{N_i=0,1} e^{-\beta(-\varepsilon-\mu)N_i} = z_{gc} = 1 + e^{\beta(\varepsilon+\mu)} \tag{S1}$$

where z_{gc} is the local grand canonical partition function, N_i is the receptor site occupation state, β is defined as the constant equal to $1/k_BT$, the k_B is Boltzmann constant ($1.380649x10^{-23}$ J K^{-1}), T is the temperature (K), -ε is the adsorption energy, and μ is the chemical potential of the adsorbed molecule.

The grand canonical partition function related to the total (Z_{gc}) N_m receptor sites is given by the following:

$$Z_{gc}=\prod_{i=1}^{N_m} z_{gc}=\left(z_{gc}\right)^{N_m}=\left(1+e^{\beta(\varepsilon+\mu)}\right)^{N_m} \tag{S2}$$

Considering that the average number of the occupied sites is given by:

$$N_0=\frac{1}{\beta}\frac{\partial}{\partial\mu}\ln\left(Z_{gc}\right)=\frac{N_m}{\beta}\frac{\partial}{\partial\mu}\ln\left(z_{gc}\right) \tag{S3}$$

The partial derivative of the local grand canonical partition function to the chemical potential is given by:

$$\frac{\partial}{\partial\mu}\ln\left(z_{gc}\right)=\beta\frac{e^{\beta(\varepsilon+\mu)}}{1+e^{\beta(\varepsilon+\mu)}} \tag{S4}$$

Substituting Equation (S4) in (S3) and considering the average adsorption energy (ε_m) and the chemical potential of the dissolved molecule (μ_d):

$$N_0=\frac{N_m}{1+(e^{-\beta(\varepsilon+\mu)})^n} \tag{S5}$$

$$N_0=\frac{N_m}{1+\left(e^{-\beta\varepsilon_m}e^{-\beta\mu_d}\right)^n} \tag{S6}$$

The average adsorption energy and the chemical potential of the dissolved molecule are related to the translation partition function as follows:

$$e^{-\mu_d\beta}=\frac{Z_v}{C_e} \tag{S7}$$

$$e^{-\varepsilon_m\beta}=\frac{Z_v}{C_{1/2}} \tag{S8}$$

Substituting Equations (S7) and (S8) onto (S6):

$$N_0=\frac{N_m}{1+\left(\frac{C_{1/2}}{C_e}\right)^n} \tag{S9}$$

Multiplying both sides of Equation (S9) by the number of molecules (n) leads to the monolayer mode with one energy site

$$Q_0=\frac{Q_m}{1+\left(\frac{C_{1/2}}{C_e}\right)^n} \tag{S10}$$

Where $C_{1/2}$ is the concentration at half-saturation (mg L^{-1}), Q_m is the adsorption capacity at the saturation (mg g^{-1}), C_e is the concentration at the equilibrium (mg L^{-1}). From the $C_{1/2}$ is possible to calculate the change in the energy adsorption according to Equation (S11):

$$\Delta E_a=-RT\ln\left(\frac{C_{1/2}}{C_e}\right) \tag{S11}$$

where ΔE_a is the adsorption energy change of one adsorbed mole (kJ mol^{-1}), R is the gas constant (kJ mol^{-1} K^{-1}), T is the temperature of the system (K).

The monolayer model with two energy sites with same and different o number of molecules considers the following: i) it is considering that a variable number of molecules N are adsorbed onto two independent types of sites, denominated as N_{m1} for the first and N_{m2} for the second, respectively. ii) same number of molecules (n) or a different number of molecules per site (n_1 and n_2), iii) it is supposed that the adsorption onto the first type depended on the energy ($-\varepsilon_1$) and onto the second type with the energy ($-\varepsilon_2$), iv) the receptor site is supposed to be found in an empty or occupied state, being represented by

N_i (0 being empty and 1 being occupied), v) a system of grand canonical partition functions equations are used to represent the two different types of sites:

$$z_{gc1}=\sum_{N_i=0,1} e^{-\beta(-\varepsilon_1-\mu_1)N_i}=1+e^{\beta(\varepsilon_1+\mu_1)} \tag{S12}$$

$$z_{gc2}=\sum_{N_i=0,1} e^{-\beta(-\varepsilon_1-\mu_2)N_i}=1+e^{\beta(\varepsilon_2+\mu_2)} \tag{S13}$$

The total grand canonical partition function equation is:

$$Z_{gc}=\prod_{i=1}^{N_m} z_{gc}=\left(z_{gc1}\right)^{N_{m1}}\left(z_{gc2}\right)^{N_{m2}}=\left(1+e^{\beta(\varepsilon_1+\mu_1)}\right)^{N_{m1}}\left(1+e^{\beta(\varepsilon_2+\mu_2)}\right)^{N_{m2}} \tag{S14}$$

Considering that the average number of the occupied sites is given by:

$$N_0=\frac{1}{\beta}\frac{\partial}{\partial\mu}\ln\left(Z_{gc}\right) \tag{S15}$$

As for the partial derivative of the total grand canonical partition function:

$$\frac{\partial}{\partial\mu}ln\left(Z_{gc}\right)=\beta\left[\frac{N_{m1}}{1+e^{-\beta(\varepsilon_1+\mu_1)}}+\frac{N_{m2}}{1+e^{-\beta(\varepsilon_2+\mu_2)}}\right] \tag{S16}$$

Substituting Equation (S16) in (S15) and considering the average adsorption energy (ε_{m1} and ε_{m2} for the site one and two respectively) and the chemical potential of the dissolved molecule (μ_d):

$$N_0=\frac{N_{m1}}{1+e^{-\beta(\varepsilon_{n1}+\mu_{d1})^{n_1}}}+\frac{N_{m2}}{1+e^{-\beta(\varepsilon_{n2}+\mu_{d2})^{n_2}}} \tag{S17}$$

The average adsorption energy for the site one and two, and the chemical potential of the dissolved molecule are related to the translation partition function as follows:

$$e^{-\mu_{d1}\beta}=\frac{Z_v}{C_e} \tag{S18}$$

$$e^{-\mu_{d2}\beta}=\frac{Z_v}{C_e} \tag{S19}$$

$$e^{-\varepsilon_{m1}\beta}=\frac{Z_v}{C_1} \tag{S20}$$

$$e^{-\varepsilon_{m2}\beta}=\frac{Z_v}{C_2} \tag{S21}$$

Substituting Equations (S18) to (S22) onto (S17):

$$N_0=\frac{N_{m1}}{1+\left(\frac{C_1}{C_e}\right)^n}+\frac{N_{m2}}{1+\left(\frac{C_2}{C_e}\right)^n} \tag{S22}$$

Multiplying both sides of Equation (S22) by the number of molecules (n) leads to the monolayer model with two energy sites and same number of molecules per site

$$Q_0=\frac{Q_{m1}}{1+\left(\frac{C_1}{C_e}\right)^n}+\frac{Q_{m2}}{1+\left(\frac{C_2}{C_e}\right)^n} \tag{S23}$$

And for different number of molecules per site:

$$Q_0=\frac{Q_{m1}}{1+\left(\frac{C_1}{C_e}\right)^{n_1}}+\frac{Q_{m2}}{1+\left(\frac{C_2}{C_e}\right)^{n_2}} \tag{S24}$$

Where C_1 and C_2 are the concentration at half-saturation for the site one and two, respectively (mg L^{-1}), Q_{m1} and Q_{m2} are the adsorption capacity at the saturation (mg g^{-1}), C_e is the concentration at the equilibrium (mg L^{-1}), n_1 and n_2 are the number of molecules adsorbed on the site one and two, respectively. From the C_1 and C_2 is possible to calculate the change in the energy adsorption for each site, according to Equations (S24) and (S25):

$$\Delta E_{a1}=-RT\ln\left(\frac{C_1}{C_e}\right) \tag{S25}$$

$$\Delta E_{a2}=-RT\ln\left(\frac{C_2}{C_e}\right) \tag{S26}$$

where ΔE_{a1} and ΔE_{a2} are the change in the adsorption energy change of one adsorbed mole for sites one and two (kJ mol^{-1}), R is the gas constant (kJ mol^{-1} K^{-1}), T is the temperature of the system (K).

For the dual-layer model with one energy site, the following formalization is given: i) it is considering that a variable number of molecules N are adsorbed onto N_m sites; ii) the receptor site is supposed to be empty or occupied by one or more molecules, being represented by N_i (0 being empty and 1 being occupied); iii) the grand canonical partition function is used to describe the phenomena for one site:

$$z_{gc} = \sum_{N_i=0,1,2} e^{-\beta(-\varepsilon-\mu)N_i} = z_{gc} = 1 + e^{\beta(\varepsilon+\mu)} + e^{2\beta(\varepsilon+\mu)} \tag{S27}$$

The grand canonical partition function related to the total (Z_{gc}) N_m receptor sites is given by the following:

$$Z_{gc} = \prod_{i=1}^{N_m} z_{gc} = \left(z_{gc}\right)^{N_m} = \left(1 + e^{\beta(\varepsilon+\mu)} + e^{2\beta(\varepsilon+\mu)}\right)^{N_m} \tag{S28}$$

Considering that the average number of the occupied sites is given by:

$$\mathrm{N_0} = \frac{1}{\beta}\frac{\partial}{\partial \mu}\ln\left(\mathrm{Z_{gc}}\right) \tag{S29}$$

As for the partial derivative of the total grand canonical partition function:

$$\frac{\partial}{\partial \mu} ln\left(Z_{gc}\right) = N_m \beta \frac{e^{\beta(\varepsilon+\mu)} + 2e^{2\beta(\varepsilon+\mu)}}{1 + e^{\beta(\varepsilon+\mu)} + e^{2\beta(\varepsilon+\mu)}} \tag{S30}$$

Substituting Equation (S29) in (S30) and considering the average adsorption energy (ε_m) and the chemical potential of the dissolved molecule (μ_d):

$$N_0 = N_m \frac{e^{\left(e^{-\beta\varepsilon_m} e^{-\beta\mu_d}\right)^n} + 2e^{2\left(e^{-\beta\varepsilon_m} e^{-\beta\mu_d}\right)^n}}{1 + e^{\left(e^{-\beta\varepsilon_m} e^{-\beta\mu_d}\right)^n} + e^{2\left(e^{-\beta\varepsilon_m} e^{-\beta\mu_d}\right)^n}} \tag{S31}$$

Substituting the correlations of the average adsorption energy and the chemical potential of the dissolved molecule (Equations (S7) and (S8)), and multiplying both of the equations by the number of molecules:

$$Q_0 = Q_m \frac{\left(\frac{C_e}{C_{1/2}}\right)^n + 2\left(\frac{C_e}{C_{1/2}}\right)^{2n}}{1 + \left(\frac{C_e}{C_{1/2}}\right)^n + \left(\frac{C_e}{C_{1/2}}\right)^{2n}} \tag{S32}$$

The dual-layer with two energy sites has a similar deduction to the dual-layer with one energy site, take into consideration: i) it is considering that a variable number of molecules N are adsorbed onto N_m sites; ii) the receptor site is supposed to be empty or occupied by one or more molecules, being represented by N_i (0 being empty and 1 being occupied);iii) the layer have different energies, being $-\varepsilon_1$ and for the first layer and $-\varepsilon_2$

for the layer above; iv) the grand canonical partition function is used to describe the phenomena for dual layer. The main difference with the dual-layer model is that is consider two energy sites, according to the following Equations:

$$e^{-\varepsilon_{m1}\beta}=\frac{Z_v}{C_1} \tag{S33}$$

$$e^{-\varepsilon_{m2}\beta}=\frac{Z_v}{C_2} \tag{S34}$$

Performing the same derivation as for the dual-layer with one energy, but the $C_{1/2}$ is exchanged for C_1 and C_2:

$$Q_0=Q_m\frac{\left(\frac{C_e}{C_1}\right)^n+2\left(\frac{C_e}{C_2}\right)^{2n}}{1+\left(\frac{C_e}{C_1}\right)^n+\left(\frac{C_e}{C_2}\right)^{2n}} \tag{S35}$$

S2. Derivation of the configurational entropy, Gibbs free energy, and energy of adsorption for the monolayer model with two energy sites and same number of molecules per site

S2.1 Configurational entropy

The configurational entropy from the physics statistics point of view is defined as follows:

$$\frac{S_a}{k_B}=ln\left(Z_{gc}\right)-\beta\frac{\partial}{\partial\beta}ln\left(Z_{gc}\right) \tag{S36}$$

where S_a is the configuration entropy (kJ mol^{-1} K^{-1}).

For the derivation of configurational entropy, the partial derivative of the β constant concerning the natural logarithm of the total grand canonical partition function. For the case of the monolayer model with two energy sites, the derivative is:

$$\frac{\partial}{\partial\beta}ln\left(Z_{gc}\right)=N_{m1}(\varepsilon_1+\mu_1)\frac{e^{\beta(\varepsilon_1+\mu_1)}}{1+e^{\beta(\varepsilon_1+\mu_1)}}+N_{m2}(\varepsilon_2+\mu_2)\frac{e^{\beta(\varepsilon_2+\mu_2)}}{1+e^{\beta(\varepsilon_2+\mu_2)}} \tag{S37}$$

Substituting Equation (S37) and (S14) onto Equation (S36) and employing the average energy adsorption energy and the chemical potential of the dissolved molecule leads to the following Equation:

$$\frac{S_a}{k_B} = N_{m1}\, ln\left(1 + e^{\beta(\varepsilon_{n1}+\mu_{d1})^{n_1}}\right) + N_{m2}\, ln\left(1 + e^{\beta(\varepsilon_{n2}+\mu_{d2})^{n_2}}\right)$$
$$-\beta\left[N_{m1}(\varepsilon_{n1}+\mu_{d1})\frac{e^{\beta(\varepsilon_{n1}+\mu_{d1})^{n_1}}}{1+e^{\beta(\varepsilon_{n1}+\mu_{d1})^{n_1}}} + N_{m2}(\varepsilon_{n2}+\mu_{d2})\frac{e^{\beta(\varepsilon_{n2}+\mu_{d2})^{n_2}}}{1+e^{\beta(\varepsilon_{n2}+\mu_{d2})^{n_2}}}\right] \quad \text{(S38)}$$

Employing Equations (18) to (22) leads to the configurational Entropy model:

$$\frac{S_a}{k_B} = N_{m1}\left[ln\left(1+\left(\frac{C_e}{C_1}\right)^n\right) - ln\left(1+\left(\frac{C_e}{C_1}\right)^n\right)\frac{\left(\frac{C_e}{C_1}\right)^n}{1+\left(\frac{C_e}{C_1}\right)^n}\right]$$
$$+N_{m2}\left[ln\left(1+\left(\frac{C_e}{C_2}\right)^n\right) - ln\left(1+\left(\frac{C_e}{C_2}\right)^n\right)\frac{\left(\frac{C_e}{C_2}\right)^n}{1+\left(\frac{C_e}{C_2}\right)^n}\right] \quad \text{(S39)}$$

S2.2 Configurational Gibbs free energy

The Gibbs free energy (G_a, Kj mol^{-1}) is represented by the following Equation:

$$G_a = \mu Q_0 \quad \text{(S40)}$$

The chemical potential is related to the translation partition function:

$$\mu = \frac{1}{\beta} ln(Z_{tr}) \quad \text{(S41)}$$

$$Z_{tr} = \frac{C_e}{\left(\frac{2\pi m}{h^2\beta}\right)^{3/2}} \quad \text{(S42)}$$

where Z_{tr} is the translation partition function, h is the Planck constant (6.62607004×10^{34} m^2 kg s^{-1}), m is the adsorbate mass (mg mol^{-1}), μ is the chemical potential (kJ mol^{-1}).

Substituting Equations (S22), (S38), and (S40) in Equation (S39):

$$G_a\beta = ln\left(\frac{C_e}{\left(\frac{2\pi m}{h^2\beta}\right)^{3/2}}\right)\left(\frac{Q_{m1}}{1+\left(\frac{C_1}{C_e}\right)^n}+\frac{Q_{m2}}{1+\left(\frac{C_2}{C_e}\right)^n}\right) \tag{S43}$$

S2.3 Internal adsorption energy

The internal energy adsorption is given by Equation (S44):

$$E_{int} = \frac{\mu}{\beta}\left(\frac{\partial}{\partial\mu}ln(Z_{gc})\right) - \frac{\partial}{\partial\beta}ln(Z_{gc}) \tag{S44}$$

Considering that the partial derivatives have been already given by Equations (S16) and (S36):

$$E_{int} = \frac{\mu}{\beta}\left(\beta\left[N_{m1}\frac{e^{\beta(\varepsilon_1+\mu_1)}}{1+e^{\beta(\varepsilon_1+\mu_1)}} + N_{m2}\frac{e^{\beta(\varepsilon_2+\mu_2)}}{1+e^{\beta(\varepsilon_2+\mu_2)}}\right]\right)$$
$$-N_{m1}(\varepsilon_1+\mu_1)\frac{e^{\beta(\varepsilon_1+\mu_1)}}{1+e^{\beta(\varepsilon_1+\mu_1)}} + N_{m2}(\varepsilon_2+\mu_2)\frac{e^{\beta(\varepsilon_2+\mu_2)}}{1+e^{\beta(\varepsilon_2+\mu_2)}} \tag{S45}$$

Similarly, as done for the Entropy, the average adsorption energy, and the chemical potential of the molecule are employed, leading to:

$$E_{int} = N_{m1}\left[\mu\frac{\left(\frac{C_e}{C_1}\right)^n}{1+\left(\frac{C_e}{C_1}\right)^n} - \frac{1}{\beta}ln\left(1+\left(\frac{C_e}{C_1}\right)^n\right)\frac{\left(\frac{C_e}{C_1}\right)^n}{1+\left(\frac{C_e}{C_1}\right)^n}\right]$$
$$+N_{m2}\left[\mu\frac{\left(\frac{C_e}{C_2}\right)^n}{1+\left(\frac{C_e}{C_2}\right)^n} - \frac{1}{\beta}ln\left(1+\left(\frac{C_e}{C_2}\right)^n\right)\frac{\left(\frac{C_e}{C_2}\right)^n}{1+\left(\frac{C_e}{C_2}\right)^n}\right] \tag{S46}$$

S2.4 Adsorption enthalpy

The adsorption enthalpy can be written as:

$$\mathrm{H_a} = \mathrm{G_a} - \mathrm{TS_a} \tag{S47}$$

Substituting the Equations for the configuration entropy and Gibbs free energy:

$$H_a = \begin{cases} \ln\left(\frac{C_e}{\left(\frac{2\pi m}{h^2\beta}\right)^{3/2}}\right)\left(\frac{Q_{m1}}{1+\left(\frac{C_1}{C_e}\right)^n}+\frac{Q_{m2}}{1+\left(\frac{C_2}{C_e}\right)^n}\right) \\ -Tk_B\begin{cases} N_{m1}\left[\ln\left(1+\left(\frac{C_e}{C_1}\right)^n\right)-\ln\left(1+\left(\frac{C_e}{C_1}\right)^n\right)\frac{\left(\frac{C_e}{C_1}\right)^n}{1+\left(\frac{C_e}{C_1}\right)^n}\right] \\ +N_{m2}\left[\ln\left(1+\left(\frac{C_e}{C_2}\right)^n\right)-\ln\left(1+\left(\frac{C_e}{C_2}\right)^n\right)\frac{\left(\frac{C_e}{C_2}\right)^n}{1+\left(\frac{C_e}{C_2}\right)^n}\right] \end{cases} \end{cases} \quad \text{(S48)}$$

S2.5 Statistical indicators

For the model evaluation, it was employed the coefficient of correlation (R^2), adjusted coefficient of determination (R^2_{adj}), minimum squared error (MSR, $(mg\ g^{-1})^2$), average relative error (ARE, %), and Bayesian Criterion Indicator (BIC)

$$R^2 = 1-\frac{\sum_{i=1}^{n}\left(q_{exp}-q_{pred}\right)^2}{\sum_{i=1}^{n}\left(q_{exp}-\bar{q}_{exp}\right)^2} \quad \text{(S49)}$$

$$R^2_{adj} = 1-\left(1-R^2\right)\frac{(n-1)}{(n-p-1)} \quad \text{(S50)}$$

$$ARE = \frac{100\%}{n}\sum_{i=1}^{n}\left|\frac{q_{exp}-q_{pred}}{q_{exp}}\right| \quad \text{(S51)}$$

$$MSR = \frac{1}{n-p}\sum_{i=1}^{n}\left(q_{exp}-q_{pred}\right)^2 \quad \text{(S52)}$$

$$BIC=nLn\left(\frac{RSS}{n}\right)+pLn(n) \quad \text{(S53)}$$

where q_{exp} is experimental adsorption capacity at the equilibrium (mg g^{-1}) and q_{pred} is the predicted adsorption capacity at the equilibrium (mg g^{-1}), n is the number of experimental data points, p is the number of parameters used in the model.

S3. Estimated parameters results

Table S1. –Steric parameters for the adsorption of methylene blue.

Models	Temperature (ºC)		
	30	40	50
MLO			
Q_m *(mg g⁻¹)*	313.3	386.2	408.2
$C_{1/2}$ *(mg L⁻¹)*	12.39	28.15	24.89
n *(dimensionless)*	0.9916	1.248	1.044
MLTS			
Q_{m1} *(mg g⁻¹)*	266.8	280.0	249.4
Q_{m2} *(mg g⁻¹)*	46.95	107.1	155.9
N_{m1} *(mg g⁻¹)*	177.4	140.9	108.5
N_{m2} *(mg g⁻¹)*	15.5	22.9	10.5
C_1 *(mg L⁻¹)*	2.291	3.240	2.825
C_2 *(mg L⁻¹)*	266.8	280.0	249.4
n *(dimensionless)*	46.95	107.1	155.9
MLTD			
Q_{m1} *(mg g⁻¹)*	255.37	248.44	252.79
Q_{m2} *(mg g⁻¹)*	59.23	150.00	150.00
N_{m1} *(mg g⁻¹)*	18.00	61.02	27.34
N_{m2} *(mg g⁻¹)*	150.0	0.757	109.6
C_1 *(mg L⁻¹)*	3.220	1.346	57.22
C_2 *(mg L⁻¹)*	1.950	4.882	3.183
n_1 *(dimensionless)*	255.37	248.44	252.79
n_2 *(dimensionless)*	59.23	150.00	150.00
DLO			
Q_m *(mg g⁻¹)*	164.5	207.0	231.5
$C_{1/2}$ *(mg L⁻¹)*	10.49	29.01	30.40
n *(dimensionless)*	0.5586	0.7131	0.5328
DLT			
Q_{m1} *(mg g⁻¹)*	164.5	207.0	231.5
C_1 *(mg L⁻¹)*	10.49	29.01	30.40
C_2 *(mg L⁻¹)*	894.4	829.9	799.9
n *(dimensionless)*	0.559	0.713	0.533

Table S2. Statistical indicators for the PSMs.

Models	Statistical indicators			
	R_{adj}^2	ARE (%)	MSE $(mg\ g^{-1})^2$	AIC
MLO	0.9982	1.008	14.28	26.87
	0.9880	3.129	150.2	48.05
	0.9789	4.405	291.1	54.01
MLTS	0.9999	0.1393	0.4651	-3.203
	0.9933	1.4969	75.40	42.59
	0.9994	0.5207	7.8354	22.22
MLT	0.9999	0.1181	0.4239	-4.428
	0.9841	2.1058	158.2	48.87
	0.9983	0.5995	17.87	29.24
DLO	0.9995	0.4913	3.742	14.82
	0.9922	2.472	97.08	44.12
	0.9888	2.994	155.5	48.36
DLT	0.9994	0.4913	4.4904	17.02
	0.9903	2.472	116.5	46.32
	0.9859	2.994	186.6	50.56

Figure S1. Speciation curves for the methylene blue.

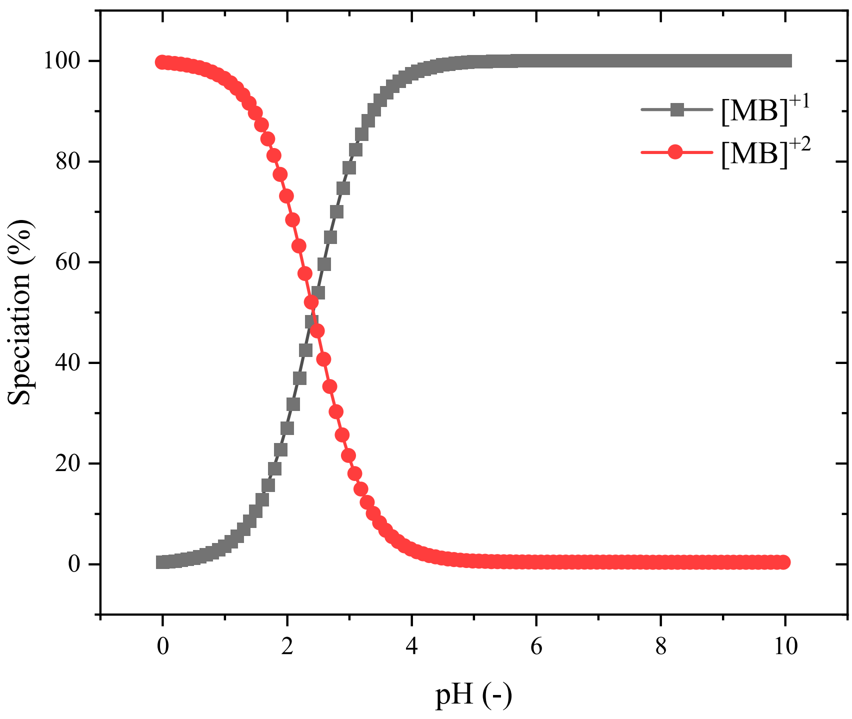

Figure S2. Molecular states for the methylene blue.

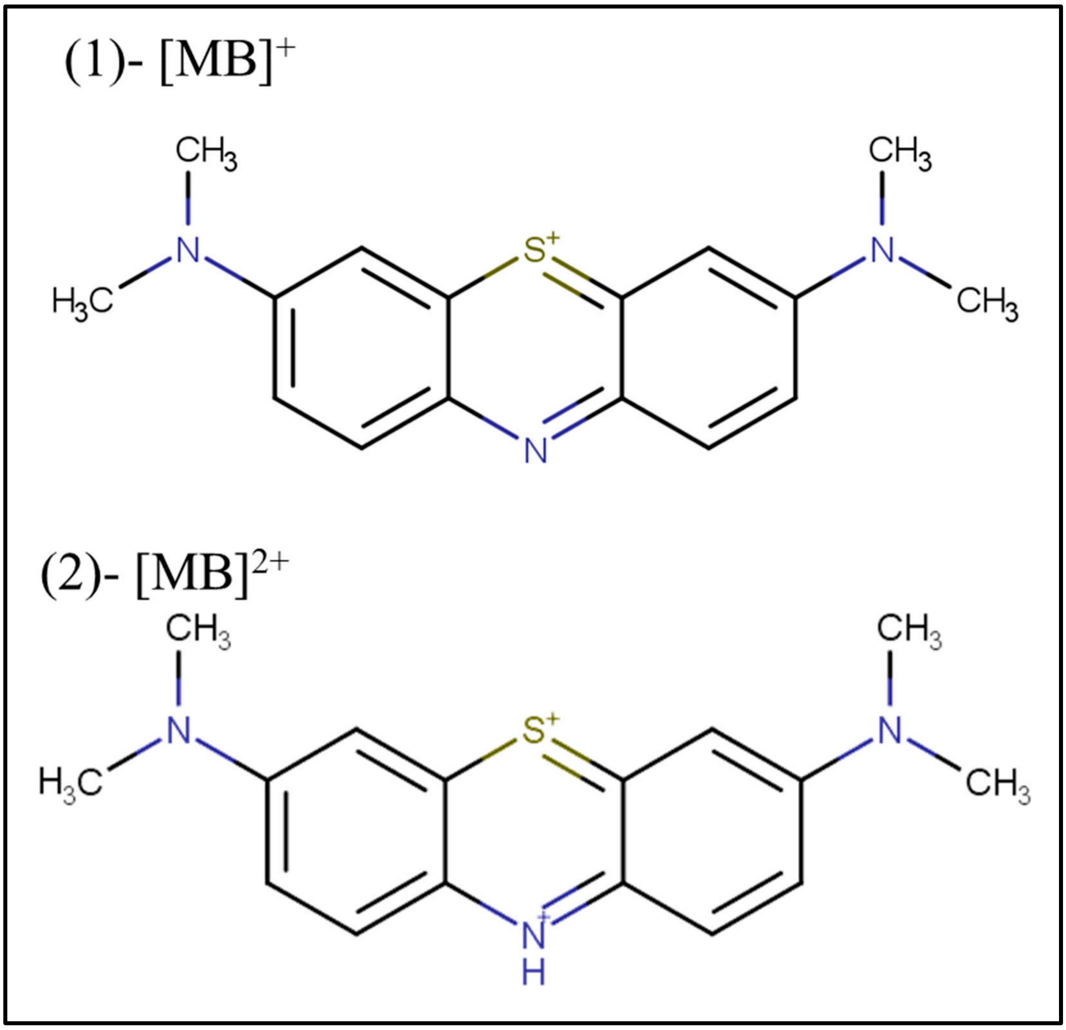

Supplement: Supplementary file 1 [file molecules-29-00412-s001.zip › molecules-2667209-supplementary.pdf]
